# Mood and neural responses to social rejection do not seem to be altered in resilient adolescents with a history of adversity

**DOI:** 10.1017/S0954579419000178

**Published:** 2020-05

**Authors:** Jessica Fritz, Jason Stretton, Adrian Dahl Askelund, Susanne Schweizer, Nicholas D. Walsh, Bernet M. Elzinga, Ian M. Goodyer, Paul O. Wilkinson, Anne-Laura van Harmelen

**Affiliations:** 1 Department of Psychiatry, University of Cambridge, Cambridge, UK; 2 Medical Research Council Cognition and Brain Sciences Unit, University of Cambridge, Cambridge, UK; 3 Institute of Cognitive Neuroscience, University College London, London, UK; 4 School of Psychology, University of East Anglia, Norwich, UK; 5 Department of Psychology, Leiden University, Leiden, the Netherlands; 6 Cambridgeshire and Peterborough NHS Foundation Trust, Cambridge, UK

**Keywords:** anterior insula, dorsal anterior cingulate cortex, mental health resilience, social rejection, social support

## Abstract

Childhood adversity (CA) increases the risk of subsequent mental health problems. Adolescent social support (from family and/or friends) reduces the risk of mental health problems after CA. However, the mechanisms of this effect remain unclear, and we speculate that they are manifested on neurodevelopmental levels. Therefore, we investigated whether family and/or friendship support at ages 14 and 17 function as intermediate variables for the relationship between CA before age 11 and affective or neural responses to social rejection feedback at age 18. We studied 55 adolescents with normative mental health at age 18 (26 with CA and therefore considered “resilient”), from a longitudinal cohort. Participants underwent a Social Feedback Task in the magnetic resonance imaging scanner. Social rejection feedback activated the dorsal anterior cingulate cortex and the left anterior insula. CA did not predict affective or neural responses to social rejection at age 18. Yet, CA predicted better friendships at age 14 and age 18, when adolescents with and without CA had comparable mood levels. Thus, adolescents with CA and normative mood levels have more adolescent friendship support and seem to have normal mood and neural responses to social rejection.

Over half of the Western population has been exposed to at least one type of childhood adversity (CA; US National Comorbidity Replication Survey; Greif Green et al., 2010). Facing adversities in childhood is a serious environmental hazard with deleterious mental health consequences across the life span (Gilbert et al., 2009; Kessler, Davis, & Kendler, 1997). Various studies have shown that CA is associated with an increased vulnerability to the development of psychopathology (Greif Green et al., 2010; Kessler et al., 2010) and that individuals with a history of CA are prone to suffer from cognitive, emotional, and social difficulties (Cicchetti, 2013; Cicchetti & Rogosch, 1997; Spinhoven et al., 2010; Walsh, Dawson, & Mattingly, 2010). For example, those exposed to CA are more likely to experience social rejection (e.g., emotional and physical bullying; van Harmelen et al., 2016). However, not all individuals who face adversity develop mental illnesses, and thus are characterized as “mentally healthy” or “resilient” (Afifi & MacMillan, 2011; Fritz, de Graaff, Caisley, van Harmelen, & Wilkinson, 2018).

Mental health following adversity is facilitated by various so-called resilience or protective factors, including biological (e.g., genes), intraindividual (e.g., distress tolerance), family (e.g., family support), and community factors (e.g., friendship support; Fritz et al., 2018; Ioannidis, Askelund, & van Harmelen, 2017; Kalisch et al., 2017). However, it is unclear what the neural mechanisms of these protective factors are (Cicchetti, 2013; Sippel, Pietrzak, Charney, Mayes, & Southwick, 2015). An improved understanding of the factors that decrease adolescents’ vulnerability to daily life stress, such as social rejection, is crucial in order to reduce the risk of mental and neural vulnerability to the development of mental illnesses after CA.

Social support significantly decreases the probability of negative mental health consequences in individuals with a history of CA. However, individuals who have been exposed to CA seem to experience less social support during adolescence and young adulthood than their peers without a history of adversity (e.g., Horan & Widom, 2015; Miller, Adams, Esposito-Smythers, Thompson, & Proctor, 2014; Sperry & Widom, 2013). The definition of social support can encompass various environmental layers, ranging from intimate/family, to friendship, to community support, up to international support networks (Sippel et al., 2015). Some studies have suggested that support from both friends and family contribute to the protective effect of social support (Horan & Widom, 2015; Runtz & Schallow, 1997; van Harmelen et al., 2016). More specifically, both friendship and family support have been found to reduce the risk of subsequent psychopathology (Dion et al., 2016; Folger & O'Dougherty Wright, 2013; Horan & Widom, 2015; Runtz & Schallow, 1997; Sperry & Widom, 2013; van Harmelen et al., 2016). However, it is as yet unknown what the mechanisms are through which social support increases resilience following CA. One potential account is that social support increases resilience by decreasing adolescents’ vulnerability to social stress, such as social rejection.

Several recent reviews consistently concluded that, at the neural level, social rejection is associated with activation in the (dorsal) anterior cingulate cortex ([d]ACC) and the (anterior) insula ([A]I; Cacioppo et al., 2013; Kawamoto, Ura, & Nittono, 2015; Wang, Braun, & Enck, 2017). Moreover, our recent study showed that in late adolescence and young adulthood, the AI and the dACC may be implicated in responsivity to social evaluation even more broadly, as those regions were similarly activated during social rejection and acceptance feedback (Dalgleish et al., 2017). The AI and the dACC are suggested to be particularly important for the detection and the appraisal of adverse social situations (Kawamoto et al., 2015). More specifically, the Insula is known to be involved in cognitive control, emotion, motivation, and pain (Wager & Feldman Barrett, 2017), whereas the dACC is associated with the evaluation and specification of control (Shenhav, Cohen, & Botvinick, 2016). Of particular importance, CA is associated with altered neural responses to social rejection (Wang et al., 2017). For example, adolescents with a history of chronic social rejection experiences in childhood displayed increased dACC and dorsal medial prefrontal cortex responsivity (van Harmelen et al., 2014; Will, van Lier, Crone, & Güroğlu, 2016), and lower dACC, dorsolateral prefrontal cortex, inferior parietal cortex, and insula cortex responsivity was observed in those with adverse loss and separation experiences in childhood (Puetz et al., 2014). As altered neural responsivity to social rejection is associated with later depressive symptoms (Masten et al., 2011), altered neural responsivity to social rejection in those with a history of CA may further increase the vulnerability to psychopathology (cf. latent vulnerability theory; McCrory & Viding, 2015).

Studies exploring the putative protective effect of social support on social rejection responsivity showed that social support is associated with decreased responsivity in the (anterior) insula (Masten, Telzer, Fuligni, Lieberman, & Eisenberger, 2012; Onoda et al., 2009) and the dACC (Eisenberger, Gable, & Lieberman, 2007; Masten et al., 2012). Thus, social support may facilitate healthy neural functioning through its impact on AI and dACC responsivity to social rejection. However, it remains unknown whether adolescent family and friendship support similarly reduces responsivity to social rejection in individuals with a history of CA.

Here, we aimed to examine whether adolescent social support reduces neural responsivity to social rejection following the exposure to CA. Due to ongoing social and neural development during adolescence (Casey, Getz, & Galvan, 2008; Crone & Dahl, 2012; Crone & Elzinga, 2015), the protective effects of social support may vary across adolescence. Therefore, we examined social support during early, as well as late, adolescence. The proposed study was conducted in a representative subsample (*N* = 55) of the longitudinal ROOTS cohort (*N* = 1,238; Goodyer, Croudace, Dunn, Herbert, & Jones, 2010). In a previous report in the larger ROOTS cohort, we found that family support mediated, but not moderated, the relationship between CA and depressive symptoms (van Harmelen et al., 2016). Accordingly, we investigated here whether early and/or late adolescent family and friendship support function as intermediate variables for the relationship between CA and (affective and/or neural) responsivity to later social rejection. The investigated ROOTS subsample only included adolescents without recent psychiatric disorder episodes at age 18, which makes it more likely that the assessment of affective and neural responsivity to social rejection is not confounded by concurrent psychopathological symptoms. We used path models to examine whether family and/or friendship support at age 14 and age 17 function as intermediate variables for the relationship between CA before age 11 and affective (i.e., mood ratings) or neural responses (i.e., AI and dACC responses) to social rejection at age 18.

## We expected that

• higher levels of CA would be associated with lower levels of social support (i.e., friendship and family support)
• higher levels of social support would be associated with lower affective (i.e., negative mood) and neural (i.e., AI and dACC) responsivity to social rejection, in both adolescents with and without CA
• and explored whether social support would additionally mediate the presumably positive relationship between CA and affective and/or neural responsivity to social rejection

## Method

### Design

Participants were recruited from the longitudinal ROOTS study (Goodyer et al., 2010). The ROOTS study has the main aim of measuring risk and resilience factors across adolescence and young adulthood, in a large population sample that is drawn from schools in Cambridgeshire. The study included 1,238 adolescents (674 girls = 54.4%, 564 boys = 45.6%). All adolescents have been assessed at the ages of 14 and 17. A detailed study description can be found in Goodyer et al. (2010). A representative subsample from ROOTS (“ROOTS MRI substudy”: *N* = 67, *M*age 18.6, *SD* = 0.67, 31 females) underwent magnetic resonance imaging (MRI) scanning at age 18. The subsample was selected based on presence versus absence of CA (see below for details) and the *5-HTTLPR* genotype (i.e., s/s or l/l homozygotes; see Walsh et al., 2012, for details). Inclusion criteria for the ROOTS MRI substudy were an adequate level of the English language and normal or corrected-to-normal vision. Exclusion criteria included a recent psychiatric disorder episode (based on the Axis 1 disorder classification of the Diagnostic and Statistical Manual of Mental Disorders IV Text Revision (DSM-IV-TR; American Psychiatric Association, 2000), any experience with unconsciousness inducing neurological traumata or recent neurological conditions, recent usage of psychotropic medication, severe learning disabilities, and metal implants. Excluding potential participants with a recent psychiatric disorder episode was based on a preliminary phone screening as well as on a more thorough mental health screening at the first in-unit assessment (i.e., using the Kiddie Schedule for Affective Disorders and Schizophrenia for School-Age Children—Present and Lifetime Version; Kaufman et al., 1997). The study was approved by the Cambridgeshire Research Ethics Committee and performed in line with Good Clinical Practice principles and the Declaration of Helsinki. All participants received monetary imbursement for their partaking.

### Sample

Fifty-nine individuals from the MRI substudy completed the Social Feedback Task in the scanner. However, for 1 participant, there were technical problems with the imaging acquisition, and 3 participants indicated that they did not believe the paradigm used. Therefore, the current analyses were conducted in 55 participants (25 females, 30 males). Thirty-two of the participants belonged to the ‘”wealthy/urban prosperity” socioeconomic status (SES) group, 14 to the “comfortably off” SES group, and 9 to the “moderate means/hard-pressed” SES group. Further sample characteristics are depicted in Table 1. The current sample did not differ from the remaining ROOTS sample in terms of age (*U* = 28, *p* = .99), gender (*U* = 36, *p* = .17), SES (*U* = 31, *p* = .59), friendship support (*U* = 24, *p* = .56), family support (*U* = 19, *p* = .24), recent negative life events (*U* = 24, *p* = .84), prior psychiatric history (*U* = 28, *p* = .88), self-esteem (*U* = 24, *p* = .93), mood (*U* = 25, *p* = .88), or *5-HTTLPR* genotype (*U* = 32, *p* = .45).

**Table 1.** Sample characteristics

	*Μ*	*SD*	Range	*N*
Age 14	14.46	0.26	13.98–14.94	53
Age 17	17.49	0.29	16.92–18.11	55
Age 18	18.50	0.60	17.33–19.92	55
Friendship support (age 14)	22.02	5.53	5.00–28.00	52
Family support (age 14)	23.04	5.72	12.00–38.00	52
Friendship support (age 17)	24.72	3.08	14.00–29.00	50
Family support (age 17)	21.92	6.10	11.00–34.00	47
IQ	107.29	9.25	83.00–124.00	52
	**Age 14**		**Age 17**	
Recent negative life events (%)	—		47 (raw count: 26, *N* = 55)	
Previous psychiatric history (%)	16 (raw count: 9, *N* = 55)		29 (raw count: 16, *N* = 55)	
Self-esteem (low, moderate, high; %)	19 low, 66 moderate, 15 high (*N* = 53)		20 low, 55 moderate, 25 high (*N* = 51)	

*Note: M*, mean. *SD*, standard deviation.

### CA

CA was assessed with the Cambridge Early Experiences Interview (CAMEEI; Dunn et al., 2011; Goodyer et al., 2010). The CAMEEI is a semistructured interview, which assesses intrafamily adverse events prior to the age of 14 (Goodyer et al., 2010). The interview was retrospectively performed with a primary caregiver, which was in 96% of the cases the biological mother. The CAMEEI was found to have an adequate interrater reliability (*n* = 48, κ = .7 to .9; Goodyer et al., 2010). In line with our previous reports on this sample (Walsh et al., 2012, 2014), presence of CA in the current sample was defined as (a) family discord, (b) sexual abuse, (c) physical abuse, and/or (d) emotional abuse before the age of 11 (see Appendix A for further details). Family discord was specified as conflict and/or incidental violence within the family, as well as lack of communication and engagement within the family (clustered in mild, moderate, and severe). Only adolescents with a history of family discord that was classified as having a significant impact on daily life (see Appendix A for details) were included in the CA group. Twenty-one of the 26 adolescents with a history of CA were exposed to family discord; 2 were exposed to family discord and potential emotional abuse; 2 were exposed to family discord, potential emotional as well as potential physical abuse; and 1 participant was primarily exposed to potential physical abuse. CA versus no-CA groups did not differ in age, gender, SES, IQ, previous psychiatric history, or *5-HTTLPR* genotype (see Table 2). The CA group did report higher depressive symptoms at age 17, but not at age 14, nor at age 18. In both groups, the minority of adolescents had psychopathological symptoms at some point in life (i.e., previous psychiatric history), yet all adolescents had no recent psychiatric disorder episode at age 18 (i.e., as this was an inclusion criterion, this ensured that the assessment of affective and neural responsivity to social rejection is unlikely to be confounded by concurrent psychopathology). Hence, at age 18 the group of adolescents with a history of CA had normative, or good, mental health, and could be considered as functioning resiliently (i.e., good mental health despite adversity; Fritz et al., 2018; Kalisch et al., 2017).

**Table 2.** Sample comparison based on CA variable

Variable	No-CA	CA	*t*/χ^2^/*z* (*df*)	*p*
*N*	29	26		
Age 14	*M* = 14.50	*M* = 14.42	1.09 (51)	.28
Age 17	*M* = 17.50	*M* = 17.47	0.42 (52)	.68
Age 18	*M* = 18.59	*M* = 18.39	1.29 (52)	.20
Gender	Male = 17 Female = 12	Male = 13 Female = 13	0.14 (1)^**b**^	.71
SES	Low = 4 Moderate = 9 High = 16	Low = 5 Moderate = 5 High = 16	–0.05^c^	.96
IQ	*M* = 107.67	*M* = 106.88	0.31 (50)	.76
Depressive symptoms (age 14)^a^	*M* = 17.11	*M* = 15.92	0.39 (38)	.70
Depressive symptoms (age 17)^a^	*M* = 11.68	*M* = 17.71	−2.13 (42)	.04*
Depressive symptoms (age 18)^a^	*M* = 9.03	*M* = 11.31	−1.06 (50)	.29
Previous psychiatric history (age 14)	No = 26 Yes = 3	No = 20 Yes = 6	0.83 (1)^**b**^	.36
Previous psychiatric history (age 17)	No = 24 Yes = 5	No = 15 Yes = 11	3.05 (1)^**b**^	.08
Parental psychopathology	No = 18 Yes = 11	No = 7 Yes = 19	5.49 (1)^**b**^	.02*
*5-HTTLPR* genotype	l/l = 18 s/s = 11	l/l = 13 s/s = 13	0.40 (1)^**b**^	.53

*Note*: CA, childhood adversity. SES, socioeconomic status. ^a^A higher score indicates a more negative mood, and a lower score indicates less negative mood. ^b^The chi-squared tests were conducted with Yates's continuity correction. ^c^As SES was split in three ordered categories, we applied the two−tailed asymptotic Cochran–Armitage test. *p < .05.

### Friendship support

The Cambridge Friendship Questionnaire (CFQ; Goodyer, Wright, & Altham, 1989; van Harmelen et al., 2017) contains eight items and was utilized to assess perceived friendship support. The self-report CFQ is based on a semistructured interview and includes the following components: satisfaction with the number of friends, frequency of contact, faithfulness of relationships, teasing, conflicts, and general satisfaction with friendship quality. Five items were rated on 4-point scale, and three items on a 6-point scale. A higher total score indicates higher satisfaction with friendships. The CFQ was found to have a good external validity, and an acceptable test–retest reliability (κ = .80; van Harmelen et al., 2017).

### Family support

The McMaster Family Assessment Device—General Functioning Scale (FAD-GF; Epstein, Baldwin, & Bishop, 1983; Miller, Epstein, Bishop, & Keitner, 1985; Ridenour, Daley, & Reich, 1999) was utilized to assess the family environment in adolescence (“family support”). The FAD-GF is a 12-item self-report questionnaire that assesses successful planning and problem solving, openness and trust, feeling accepted as well as warmth of the family environment. All items were rated on 4-point scale and a higher total score indicates a higher level of family support. The FAD adequately differentiates between appropriate and inappropriate family functioning and was found to have an acceptable test–retest reliability (Epstein et al., 1983; Miller et al., 1985; Ridenour et al., 1999).

### Descriptive measures

Details of all descriptive measures can be found in Appendix B.

• ***SES*** was assessed with the ACORN, A Classification of Residential Neighborhoods (http://www.caci.co.uk; Morgan & Chinn, 1983).
• ***Intelligence (IQ)*** was assessed with the vocabulary and block design subtests of the Wechsler Abbreviated Scale of Intelligence (Wechsler, 1999).
• ***Recent negative life events*** were assessed with the Life Events Questionnaire (adapted from Goodyer, Herbert, Tamplin, & Altham, 2000; Walsh et al., 2012).
• ***Current and past psychiatric diagnosis*** was assessed with the Kiddie Schedule for Affective Disorders and Schizophrenia for School-Age Children—Present and Lifetime Version (Kaufman et al., 1997).
• ***Self-esteem*** was assessed with the Rosenberg Self-Esteem Scale (Rosenberg, 1965).
• ***Depression symptoms*** were measured with the Mood and Feeling Questionnaire (Messer, Angold, & Costello, 1995).
• ***5-HTTLPR genotype*** was retrieved from saliva samples (Walsh et al., 2012, 2014).
• ***Parental psychopathology*** was assessed with the MINI Mental State Examination (Sheehan et al., 1998).

### Functional MRI (fMRI) Social Feedback Task

**Figure 1.**
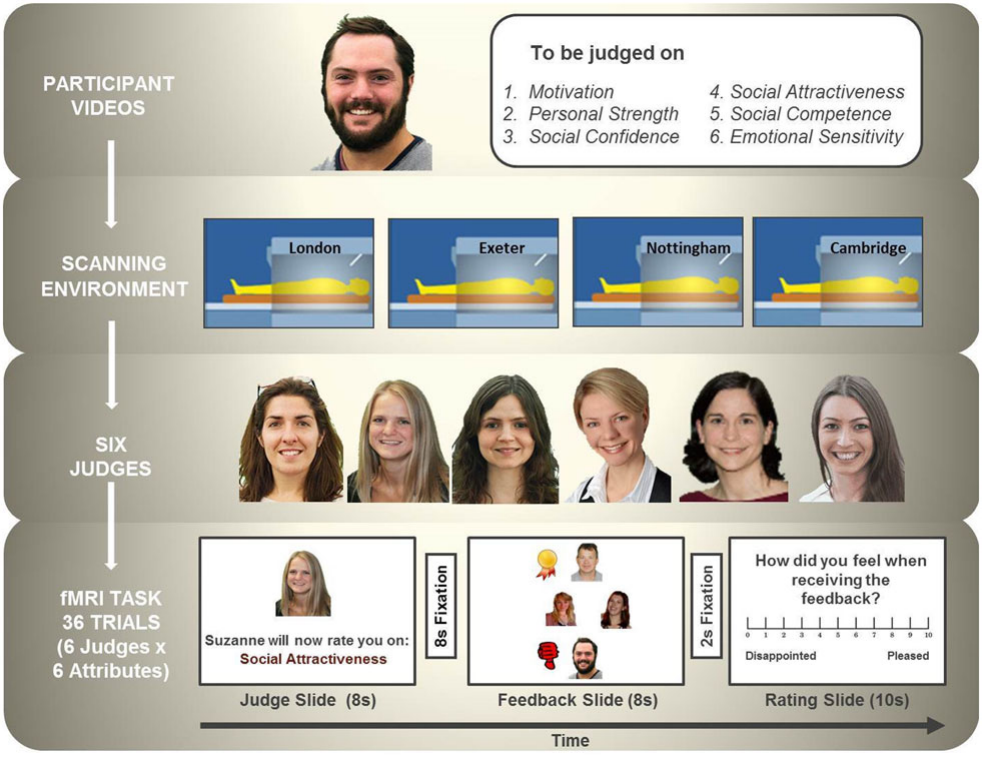
Procedure of the Social Feedback Task (with permission, adapted from Dalgleish et al., 2017; Scientific Reports; can be retrieved from https://doi.org/10.1038/srep42010; information regarding the publishing license of the original figure, and information regarding modifications we applied can be found in Appendix C).

The fMRI Social Feedback Task was set up as a competition game, in which the participants were told that they could win the game when being successful in impressing a team of six judges during all three rounds of the competition (see Figure 1; Dalgleish et al., 2017). Participants were instructed that they had to compete against three other players, and that in each round of the competition, one player would be excluded. In addition, participants were informed that they would be connected via internet to the three competitors, all being scanned at the same time at different places in the United Kingdom. In reality, the competition consisted of only one round in which each participant was rejected. During the first (and only) round of the competition, the participants had to record a video in which they should introduce themselves and their major goals and accomplishments. Beforehand, all participants were provided with one example video of a “prior” player and were told that their video would be judged on six social success variables (i.e., motivation, personal strength, social confidence, social attractiveness, social competence, and emotional sensitivity) by a team of six adult judges, being trained in video evaluation (Figure 1). Based on the video, they were told that they were either excluded or could proceed to the following (“nonexisting”) round. To decrease potential skepticism, the participants were shown photos of the team of judges and were informed that the judges were located at another research site, receiving all videos online. During the fMRI scan, the participants eventually received the judges’ feedback for their videos stating who of the four competitors was best, moderate, and worst on each of the six social success variables. The participants received the feedback from each judge on each social success variable separately, resulting in 36 feedback slides (6 judges × 6 social success variables). Each participant received 12 “best” ratings (i.e., positive), 12 “moderate” ratings (i.e., neutral), and 12 “worst” ratings (i.e., negative), while the order of the social success variables and the judges was counterbalanced. After each of the 36 ratings, the participants were asked to indicate their mood state on an 11-point Likert scale, which functioned as a measure for affective responses to rejection and acceptance feedback. To increase the authenticity of the competition, the participants additionally had to judge the videos of the three other players, by applying the same six social success variables. Finally, the participants were informed that five of the six judges rated their video generally as “worst,” and one as “moderate,” leading to the exclusion from the competition. After scanning, a manipulation check was performed to control for the authenticity of the competition, and afterward participants were debriefed (Dalgleish et al., 2017). In the current study we focused on the responsivity contrast between “worst” (i.e., negative) and “moderate” (i.e., neutral) feedback ratings: “negative more than neutral” contrast.

### fMRI image acquisition

Functional MRI data was collected with a 3-Tesla scanner (Tim Trio unit, built by Siemens, Germany). We utilized a head coil gradient set and assessed T1-weighted images with a voxel size resolution of 1 × 1 × 1 mm. We additionally assessed blood oxygen level dependent signal contrast sensitive echo-planar T2*-weighted images (EPI), which consisted of 48 sagittal slices, being 3 mm thick and having a voxel size resolution of 3 × 3 × 3 mm (repetition time = 2000 ms, echo time = 30 ms, flip angle = 78 degrees, field of view = 192 mm; Dalgleish et al., 2017).

### Image preprocessing

Functional MRI data preprocessing was performed with the statistical parametric mapping (SPM8) software, and to prevent equilibration related errors, the first five volumes were not included in the analysis. To remediate potential head movement artifacts, rigid body transformations were utilized, using the first scan as a realignment reference. To control for putative slice timing differences, a slice scan time correction was applied to the echo planar T2*-weighted images, using sinc interpolation. The FieldMap toolbox was used to calculate phase differences between the images, being assessed at the short and the long echo time, based on which field maps were established and unwrapped. Echoplanar T2* imaging parameters as well as field map parameters were utilized to identify distortions in the T2*-weighted images, which were corrected through inverse voxel displacement. (Non)linear transformations and spatial Gaussian kernel smoothing (8-mm full width at half maximum) were applied to the echo planar T2*-weighted as well as T1-weighted images, which were spatially normalized to the structural standard space of the Montreal Neurological Institute template and coregistered. Furthermore, proportional scaling and high-pass temporal filtering (with a cutoff value of 128 s) were conducted to eliminate global changes and low-frequency signal drifts (Dalgleish et al., 2017).

### fMRI data analysis and results

General linear models were used to calculate the participants’ neural activation during exposure to the 36 judge feedbacks and the belonging 36 mood state ratings. Due to the three different judge feedback options (best, moderate, and worst), an epoch-related statistical model was used to establish activation for each feedback option and the belonging mood state ratings. Activations were mean-corrected and convolved with a canonical hemodynamic response function. Six head movement parameters, derived from spatial realignment corrections, were included in the multiple linear regression models as covariates. For the below analyses we used the “negative more than neutral” responsivity contrast, which was family-wise error corrected (FWE; whole-brain, voxel-wise threshold of *p* < .05; Dalgleish et al., 2017). As a previous report on this sample (Dalgleish et al., 2017) found that the “negative more than neutral” contrast revealed a significant responsivity in the left AI and the bilateral dACC, we restricted our analyses to those two brain areas. We defined a 10-mm sphere around the peak voxels of the AI (x = –28, y = 16, z = –12 mm) and the dACC (x = 2, y = 32, z = 24 mm) and extracted the time course of activity for each region for each participant. These time courses were used for subsequent analyses.

### Current analyses

All analyses were conducted in R (R Core Team, 2017) with the Lavaan package (Rosseel, 2012), using a full information maximum likelihood (FIML) estimation approach. The FIML algorithm does not exclude missing values and establishes case-wise maximum likelihood functions, making use of all available information (Enders & Bandalos, 2001). Given that our data contained missing values, as well as deviations from normality, we utilized a robust estimator (MLR), which can calculate robust standard errors and scaled test statistics despite incomplete data (Rosseel, 2012).

**Figure 2.**
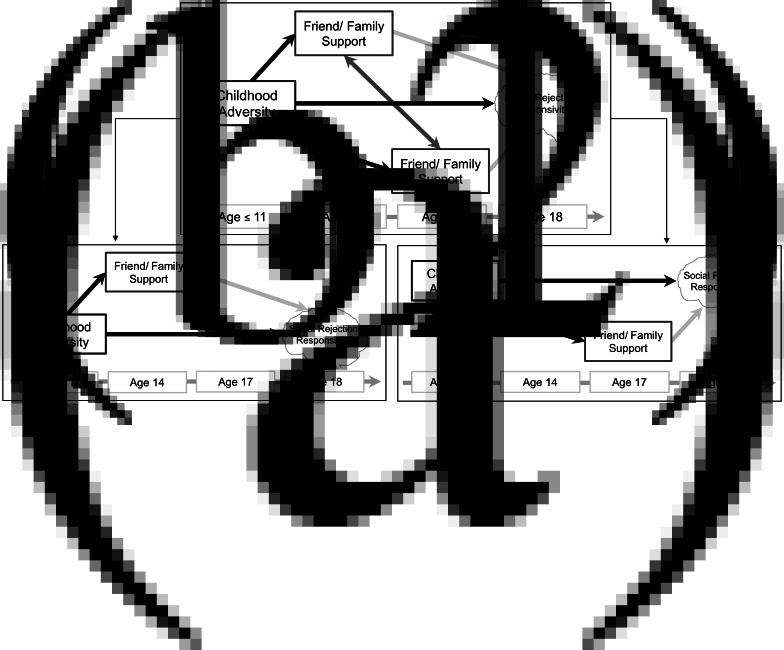
Models being tested in the below analyses. Model (a) is a path model including intermediate variables for support at age 14 and at age 17, separately for family and friendship support. The two (b) models are exploratory follow-up models and include intermediate support variables (either family or friendship support) for either age 14 (b1) or age 17 (b2), to increase the power of the analyses. Predictive paths are indicated with one-sided arrows. Correlations are indicated by two-sided arrows.

To investigate whether family and/or friendship support function as intermediate variables for the relationship between CA and responses to social rejection (affective or neural [dACC or AI] responses), we ran six path models. In each model, CA was specified as the independent variable, family support (or friendship support) at the ages of 14 and 17 were specified as intermediate variables, and responses to social rejection feedback (affective or neural [dACC or AI] responses) were specified as the dependent variable (see Figure 2a). As we were not interested in the path from age 14 to age 17 friendships or age 14 to age 17 family support, these variables were specified to covary with each other (yet, all below findings remained when age 14 *predicted* age 17 friendships or family support). To increase the power of the investigated models, we reestablished the models while only including one intermediate support variable (see Figure 2b). Along those lines, we also explored whether family and/or friendship support (at age 14 and/or 17) mediate the relationship between CA and affective or neural responses to social rejection. Standard errors of indirect and total effects were calculated according to the delta method (Rosseel, 2012; Sobel, 1982).

## Results

### Affective and neural responses to social rejection

**Figure 3.**
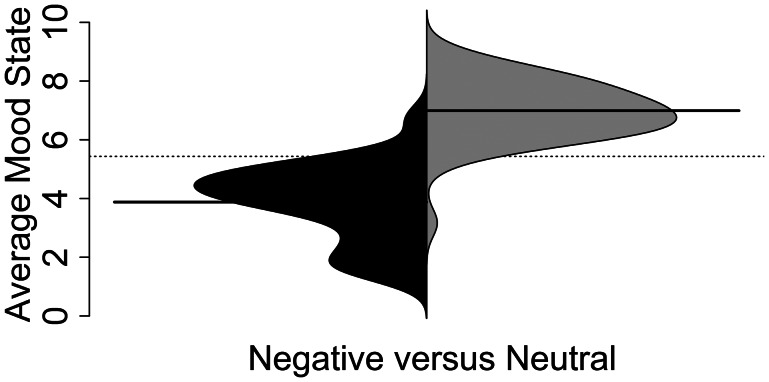
Average mood state ratings after negative and neutral feedback, during the Social Feedback Task. The left side of the bean plot (black) depicts the mood state distribution for negative and the right side (gray) for neutral social feedback. Horizontal lines represent means (continuous = feedback condition means; dotted = total mean).

In a previous report on this sample, Dalgleish et al. (2017) showed that the “negative more than neutral” contrast revealed a significant responsivity in the left AI (*z* = 4.97, *p* < .05 FWE corrected) and the bilateral dACC (*z* = 4.81, *p* < .05 FWE corrected). No other regions were activated at this threshold (see, for details, Dalgleish et al., 2017). Mood state ratings were in line with the fMRI results, given that “negative” judge feedback was experienced as more disturbing than “neutral” judge feedback, *t* (54) = –13.33, *p* < .001 (see Figure 3).

### Does adolescent friendship support function as an intermediate variable for the relationship between CA and later responses to social rejection?

Our findings showed that CA is associated with less negative mood responses to social rejection feedback, albeit this was a weak relationship (Table 3). CA was not related with AI or dACC responses to social rejection feedback. Furthermore, CA predicted *higher* levels of friendship support at age 14, but did not predict friendship support at age 17. Friendship support at age 14 was strongly associated with friendship support at age 17. However, neither friendship support at age 14, nor at age 17, predicted affective responses to social rejection feedback. Similarly, neither friendship support at age 14, nor at age 17, predicted AI or dACC responses to social rejection feedback. These results were confirmed by single follow-up mediation models, which showed that both friendship support variables did not mediate the relationship between CA and responses to social rejection feedback (i.e., affective and neural). In contrast to the significant effect of CA on friendship support at age 14 (Mean *R*^2^ = .09), the effect of CA on friendship support at age 17 was nonsignificant and negligible (Mean *R*^2^ = .03). Furthermore, the effect of CA and friendship support on mood was marginal and small (Mean *R*^2^ = .07), whereas the same effect on the brain was not only nonsignificant but also negligible (*R*^2^ = .03).

**Table 3.** Results paths and mediation models for friendship support

Indep. Var.	Dep. Var.	Est	*SE*	*z*	*p* (>|*z*|)	CI low	CI up
*Affective response to social rejection feedback*							
CA	Affective response	–.28	.14	−1.99	.05*	–.55	–.01
Friendships age 14	Affective response	.10	.17	0.60	.55	–.23	.43
Friendships age 17	Affective response	.02	.14	0.13	.90	–.26	.29
CA	Friendships age 14	.29	.13	2.24	.03*	.04	.54
CA	Friendships age 17	.16	.13	1.18	.24	–.10	.42
Friendships age 14	Friendships age 17	.40	.17	2.46	.01*	.08	.73
Indirect effect: Friendships age 14		.04	.05	0.74	.46	–.06	.13
Indirect effect: Friendships age 17		.01	.02	0.55	.58	–.03	.05
*AI response to social rejection feedback*							
CA	AI response	.03	.13	0.23	.82	–.22	.27
Friendships age 14	AI response	–.05	.21	−0.22	.83	–.45	.36
Friendships age 17	AI response	.14	.20	0.72	.47	–.24	.52
CA	Friendships age 14	.29	.13	2.20	.03*	.03	.54
CA	Friendships age 17	.16	.13	1.24	.22	–.09	.42
Friendships age 14	Friendships age 17	.41	.17	2.44	.02*	.08	.73
Indirect effect: Friendships age 14		.01	.05	0.25	.80	–.09	.12
Indirect effect: Friendships age 17		.02	.03	0.89	.38	–.03	.08
*dACC response to social rejection feedback*							
CA	dACC response	–.01	.12	−0.08	.94	–.24	.22
Friendships age 14	dACC response	–.38	.22	−1.73	.08	–.80	.05
Friendships age 17	dACC response	.21	.16	1.33	.18	–.10	.52
CA	Friendships age 14	.27	.13	2.09	.04*	.02	.53
CA	Friendships age 17	.16	.13	1.22	.22	–.10	.42
Friendships age 14	Friendships age 17	.41	.17	2.48	.01*	.09	.73
Indirect effect: Friendships age 14		–.08	.04	−1.70	.09	–.16	.01
Indirect effect: Friendships age 17		.01	.02	0.54	.59	–.03	.05

*Note*: Indep. Var., independent variable. Dep. Var., dependent variable. *SE*, standard error. CA, childhood adversity. AI, anterior insula. dACC, dorsal anterior cingulate cortex. *p < .05.

### Does adolescent family support function as an intermediate variable for the relationship between CA and later responses to social rejection?

In line with the findings for friendship support, CA was marginally associated with lower negative affective responses to social rejection feedback (Table 4). CA was not related to AI and dACC responses to social rejection feedback, and did not predict family support at ages 14 and 17. Family support at age 14 was strongly associated with family support at age 17. In contrast to our assumption, family support at age 14 and age 17 did not predict affective and AI responses to social rejection feedback. Yet, family support at age 14 was marginally associated with lower dACC responsivity, whereas family support at age 17 was marginally associated with increased dACC responsivity. Most results remained unchanged when tested separately for the support variables; however, neither family support at age 14 nor at age 17 was significantly associated with dACC responsivity to social rejection feedback. Moreover, both family support variables did not mediate the relationship between CA and responses to social rejection feedback (i.e., affective and neural). Along those lines, the effect of CA on family support at age 17 (Mean *R*^2^ = .06) and the effect of CA and family support on mood (Mean *R*^2^ = .06) did both not reach significance and had small effects. Moreover, the effect of CA on family support at age 14 (Mean *R*^2^ = .03) and the effect of CA and family support on the brain (Mean *R*^2^ = .015) were both not only nonsignificant but had negligible effects.

**Table 4.** Results paths and mediation models for family support

Indep. Var.	Dep. Var.	Est	*SE*	*z*	*p* (>|*z*|)	CI low	CI up
*Affective response to social rejection feedback*							
CA	Affective response	–.25	.13	−1.86	.06	–.51	.01
Fam. sup. age 14	Affective response	–.02	.17	−0.12	.90	–.35	.31
Fam. sup. age 17	Affective response	–.01	.19	−0.06	.96	–.38	.36
CA	Fam. sup. age 14	–.16	.14	−1.14	.26	–.42	.11
CA	Fam. sup. age 17	–.22	.14	−1.54	.12	–.49	.06
Fam. sup. age 14	Fam. sup. age 17	.47	.16	2.92	.00*	.16	.79
Indirect effect: Fam. sup. age 14		.01	.03	0.38	.70	–.04	.06
Indirect effect: Fam. sup. age 17		–.01	.04	–0.14	.89	–.09	.07
*AI response to social rejection feedback*							
CA	AI response	.04	.13	0.29	.78	–.22	.30
Fam. sup. age 14	AI response	.06	.17	0.37	.71	–.27	.40
Fam. sup. age 17	AI response	–.05	.22	−0.22	.82	–.49	.39
CA	Fam. sup. age 14	–.16	.14	−1.13	.26	–.43	.12
CA	Fam. sup. age 17	–.22	.14	−1.56	.12	–.49	.06
Fam. sup. age 14	Fam. sup. age 17	.47	.16	2.93	.00*	.16	.79
Indirect effect: Fam. sup. age 14		–.01	.03	−0.42	.68	–.06	.04
Indirect effect: Fam. sup. age 17		.00	.04	0.04	.97	–.08	.08
*dACC response to social rejection feedback*							
CA	dACC response	–.05	.12	−0.42	.68	–.29	.19
Fam. sup. age 14	dACC response	–.28	.14	−2.01	.05*	–.55	–.01
Fam. sup. age 17	dACC response	.31	.16	1.98	.05*	.00	.62
CA	Fam. sup. age 14	–.15	.14	−1.12	.27	–.42	.12
CA	Fam. sup. age 17	–.22	.14	−1.62	.11	–.50	.05
Fam. sup. age 14	Fam. sup. age 17	.48	.16	2.98	.00*	.16	.79
Indirect effect: Fam. sup. age 14		.02	.02	0.89	.37	–.03	.07
Indirect effect: Fam. sup. age 17		–.04	.04	−1.04	.30	–.12	.04

*Note*: Indep. Var., independent variable. Dep. Var., dependent variable. *SE*, standard error. CA, childhood adversity. Fam. sup., family support. AI, anterior insula. dACC, dorsal anterior cingulate cortex. *p < .05.

### Exploratory analyses: Neural responses

We additionally tested whether family and/or friendship support (separately) have *immediate* effects on social rejection responses (i.e., cross-sectional models). To this end, we examined whether friendship and family support at age 18 function as intermediate variables for the relationship between CA and neural responsivity to social rejection at age 18 (i.e., AI and dACC). In line with the above results, the analyses showed that CA was associated with a higher level of friendship support at age 18, but neither family nor friendship support at age 18 mediated the relationship between CA and neural responsivity to social rejection (see Appendix D).

The Social Feedback Task revealed not only significant main effects in the AI and the dACC for the contrast “negative more than neutral” but also for the contrast “positive more than neutral,” reflecting social *acceptance* responsivity (left AI: x = –28, y = 16, z = –12, *k*-voxel = 85, *z* statistic = 5.85, *p* < .05, FWE corrected; bilateral dACC: x = 0, y = 32, z = 24, *k*-voxel = 1218, *z* statistic = 6.57, *p* < .05, FWE corrected; for details, see Dalgleish et al., 2017). Therefore, we additionally explored whether CA and family and friendship support have effects on social *acceptance* responsivity. However, in line with the results for social *rejection* responsivity, we revealed neither an effect of CA nor an effect of friendship and/or family support on neural social *acceptance* responsivity (corrected for CA; AI: Mean *R*^2^ = .03; dACC: Mean *R*^2^ = .014). In line with the previous findings, we again found that adolescents with a history of CA have on average a higher level of adolescent friendship support at age 14 (Mean *R*^2^ = .09).

### Exploratory analyses: Gender effects

Our sample size did neither allow for examining gender as a group effect, nor as a covariate. Therefore, we explored the effects of gender through correlating CA, social support, and social rejection responsivity variables with each other, separately for males and females. For female participants, CA was associated with a significantly higher amount of friendship support at age 14 (*r* = .41; 95% confidence interval, CI [0.02, 0.69]), as well as a significantly lower amount of family support at age 17 (*r* = –.52, 95% CI [–0.76, –0.13]; see Table 5). In contrast for male participants, CA was neither significantly associated with friendship support at age 14 (*r* = .22, 95% CI [–0.17, 0.55]), nor with family support at age 17 (*r* = –.16, 95% CI [–0.53, 0.26]). Moreover, for females, CA was not associated with negative mood levels (*r* = .23, 95% CI [–0.18, 0.57]), whereas for males CA was strongly associated with a lower negative mood level during social rejection (*r* = –.49, 95% CI [–0.72, –0.15]). None of the correlational results suggested significant gender-specific findings with regard to neural responses (full correlation tables, separately for gender as well as for the overall sample, can be found in Appendix E). Hence, our post hoc explorations seemed to indicate that CA may impact the role of social support as well as affective responses to rejection differently in males and females.

**Table 5.** Post hoc exploratory correlational analyses for potential gender effects

		Females				Males				Differences			
		CA	AI	ACC	Mood	CA	AI	ACC	Mood	CA	AI	ACC	Mood
	**CA**		−0.16	−0.12	0.23		0.21	−0.04	−0.49*		−0.37	−0.08	0.72
**Friend** **support**	**Age 14**	0.41*	−0.05	−0.30	0.06	0.22	0.12	−0.24	0.00	0.19	−0.17	−0.06	0.06
	**Age 17**	0.31	0.25	0.01	0.16	0.06	0.01	0.07	−0.08	0.25	0.24	−0.06	0.23
	**Age 18**	0.29	−0.07	−0.12	−0.03	0.21	−0.05	−0.05	0.14	0.08	−0.03	−0.07	−0.17
**Family support**	**Age 14**	−0.34	0.15	−0.04	−0.07	−0.02	−0.06	−0.17	0.04	−0.32	0.21	0.12	−0.10
	**Age 17**	−0.52*	0.19	0.13	0.11	−0.16	−0.18	0.27	0.12	−0.36	0.37	−0.13	−0.01
	**Age 18**	−0.26	0.05	0.08	0.11	−0.31	0.09	0.11	0.38*	0.05	−0.04	−0.02	−0.27

*Note*: CA, childhood adversity. AI, anterior insula. ACC, anterior cingulate cortex. Mood represents negative (vs. neutral) mood responsivity during social rejection. *p < .05.

## Discussion

We showed that when adolescents with a history of CA have comparable mood levels as adolescents without CA (i.e., at ages 14 and 18), adolescents with CA have higher levels of friendship, but not family, support. Yet, in contrast to our hypothesis, social support (i.e., family and friendship support) at ages 14 and 17 was not associated with lower negative mood or neural responsivity to social rejection at age 18. Moreover, adolescents with CA did not seem to have altered neural (i.e., AI and dACC) and at best marginally altered mood responses to social rejection at age 18, when they were characterized by mental health resilience. This suggests that adolescents with CA have normal neural responses as well as normal, or perhaps even less negative, mood responses to social rejection, when they are mentally healthy.

The notion that individuals who have been exposed to CA experience less social support during adolescence and young adulthood than their peers without a history of adversity has sound support in the resilience literature (Horan & Widom, 2015; Miller et al., 2014; Runtz & Schallow, 1997; Sperry & Widom, 2013; van Harmelen et al., 2016). Yet, our result partially differed from this notion, as we found that CA did not predicted adolescent family support at age 14, at age 17, or at age 18. Moreover, we found that CA did not predict friendship support at age 17, but was associated with higher levels of adolescent friendships at ages 14 and 18. At ages 14 and 18, our sample of adolescents with CA reported similar levels of depressive symptoms as those without CA, whereas at age 17, the CA adolescents had on average higher depressive symptoms than adolescents without CA. Thus, our findings showed that when adolescents with and without CA have comparable mood levels, adolescents with CA have higher levels of friendship support. Therefore, one may speculate that not necessarily a history of CA (on its own) may influence the level of quality and quantity of adolescent friendships, but there may be a more complex interplay between mood levels and the level of adolescent friendships subsequent to CA.

As mental health resilience refers to the absence of mental health problems despite a history of adversity (Fritz et al., 2018; Kalisch et al., 2017), our CA sample is characterized by concurrent mental health resilience at the time of the social rejection assessment. Therefore, the nature of our CA variable in combination with solely selecting resilient 18-year-old CA adolescents may be another reason why CA was associated with higher levels of friendship support. That is, selecting resilient 18-year-olds, with a history of mild to moderate family adversity, may have led to the overinclusion of those with CA who received and/or perceived more friendship support in early adolescence. Our post hoc explorations of gender effects suggest that in females family-related adversity may impact predominantly on social relations, potentially resulting in higher friendship and lower family support, whereas in males family-related adversity appears to be associated with less negative mood in response to social rejection. However, as our sample size does not allow for a more complex exploration of gender effects, such conjectures remain to be tested in larger future studies.

We further found that (a) affective responses to negative rejection feedback were significantly lower than responses to neutral rejection feedback. Yet, (b) CA only marginally predicted affective responses to social rejection feedback (i.e., lower negative mood responses). Similarly, Will et al. (2016) as well as van Harmelen et al. (2014) showed that (a) social rejection is associated with negative mood responses, but (b) negative mood responses to social rejection are not specific to adolescents with a history of chronic social rejection. Thus, mood levels seem to be lower during social rejection, when compared to positive or neutral social interactions, regardless of CA exposure. Along those lines, our findings seemed to suggest that a history of CA may rather tend to go together with less negative mood responses to social rejection. This conjecture is consistent with a previous report on emotion regulation capacity in this sample (Schweizer et al., 2016), which revealed that at age 18 mentally healthy adolescents with CA are more efficient in emotion regulation than mentally healthy adolescents without CA (Schweizer et al., 2016). Therefore, enhanced emotion regulation capacity may explain why CA adolescents seemed to have normal, or perhaps even less negative, mood responses to social rejection.

Different forms of CA are found to be differentially associated with insula and dACC responsivity to social rejection, with some forms of CA even having an opposite association sign (e.g., increased dACC responsivity in adolescents with a history of chronic social rejection compared to decreased dACC responsivity in adolescents with adverse loss and separation experiences in childhood; Puetz et al., 2014; Will et al., 2016). Our data showed that CA in concurrently resilient adolescents does not predict neural (i.e., AI and dACC) responses to social rejection. Moreover, the effects were not only nonsignificant but also of a negligible size. As our CA group included various types of CA, it may have been the case that participants with chronic social rejection experiences had higher neural responses and participants with adverse loss and separation experiences had lower neural responses to social rejection, which may have canceled each other out (i.e., leading on average to similar levels of AI and dACC responses to social rejection for participants with and without a history of CA). In our study (a) social rejection by peers was not assessed, (b) none of the CA participants was adopted or in foster care, and (c) only 4 of the 26 participants with CA had a history of childhood emotional maltreatment. Therefore, we did not have enough information to disentangle potentially differing effects of rejection, and adverse loss and separation, experiences on social rejection responsivity. However, the enhanced emotion regulation capacity of CA adolescents in our sample was supported not only on the affective but also on the neural level (Schweizer et al., 2016), and thus may be an alternative explanation for our finding that CA was not associated with an increase in neural responses to social rejection.

Contrary to our hypothesis, we also did not find evidence for social support reducing later affective or neural (i.e., AI and dACC) responses to social rejection, and most of the revealed effects were not only nonsignificant but also noticeably small. The literature showed that different forms of social support, that is (a) emotionally supportive texts, (b) social interaction quality, and (c) friendship interaction frequency and duration, are associated with decreased social rejection responsivity in either the AI, the dACC, or both (Eisenberger et al., 2007; Masten et al., 2012; Onoda et al., 2009). One may speculate that our study lacked protective effects of social support, due to the developmental phases that were studied. For *family support*, this conjecture would be in line with previous findings, showing that family support appears to lower stress responsivity during childhood but not during adolescence (Hostinar, Johnson, & Gunnar, 2015). Similarly, maternal support was found to reduce unfavorable affect-related behavior and neural responses in healthy children, but not in healthy adolescents (Gee et al., 2014). Thus, whereas family support may reduce unfavorable affective and neural responses in childhood, our findings suggest that adolescent family support does not improve affective or neural responses to social rejection at age 18. For *friendship support* a lack of protective effects due to the studied developmental phases is unlikely. Masten et al. (2012) showed that higher levels of friendship interactions at age 18 are associated with lower AI and dACC responsivity to social rejection at age 20 (Masten et al., 2012), which suggests lasting protective effects of adolescent friendship support on social rejection responsivity. In sum, our findings suggest that mood and neural (AI and dACC) responses to social rejection, in mentally healthy 18-year-old adolescents, do not seem to be altered by a CA history and/ or the level of adolescent family and friendship support.

Critics may rightfully argue that the statistical power of the tested models was limited by our sample size (MacKinnon, Fairchild, & Fritz, 2007; Wolf, Harrington, Clark, & Miller, 2013), and the current findings should therefore be interpreted considering this limitation. To determine the effect size that would have enabled us to find effects from CA on support variables (*a* path) and from support variables on mood and/or brain responses (corrected for the effect of CA; *b* path), we performed post hoc sensitivity analyses (linear regression effects in G*Power; effect sizes were interpreted along Cohen's guidelines; see Faul, Erdfelder, Lang, & Buchner, 2007). We revealed that with our sample size (*M* sample size = 53 [ranging from 47 to 55 observations per variable], an α of .05 and a power of .80), we would have been able to detect moderate effects (*a* path: *f*^2^ = .154; *b* path: omnibus effect of *f*^2^ = .193 or *R*^2^ increase in variance explained of *f*^2^ = .154). Thus, as clinically relevant moderate path effects should have been detected, we believe that our conclusion, that resilient adolescents with a history of CA seem to have normal mood and neural response to social rejection, is warranted. That said, it needs to be acknowledged that power was predominantly limited for the indirect (mediation) effects (Fritz & MacKinnon, 2007; MacKinnon et al., 2007). However, as our findings revealed that (a) in none of the models both the *a* and the *b* paths were significant, and that (b) in most of the models at least one of the two path coefficients had a small effect, we believe that the null findings for the indirect (mediation) effects are the result of nonsignificant path effects. In sum, a higher sample size would have been desirable, and would have increased the chance to detect small path effects. However, this was beyond the aim of the current research.

In addition to investigating the social support variables as potential intermediate resilience mechanisms, they could also have been examined with moderation analyses. Moderation analyses would have tested whether social support has a stronger effect on social rejection responsivity for adolescents with compared to adolescents without CA (Baron & Kenny, 1986; Fritz et al., 2018). Theoretically, post hoc moderation analyses would have been highly interesting in the studied context. However, as (a) neither the main effect of CA, nor the main effect of the support variables on brain responses to social rejection revealed significance, and as (b) power analyses indicated that our sample size would not have been sufficient to detect interaction effects (see for details Appendix F), we did not perform post hoc moderation analyses.

Another potential limitation may be the rather small voxel size area for social *rejection* responsivity. Yet, the AI and dACC main effect areas for social *acceptance* responsivity (AI: *k*-voxels = 85; dACC: *k*-voxels = 1218) were notably larger than the main effect areas for social *rejection* responsivity (AI: *k*-voxels = 9; dACC: *k*-voxels = 19), and as we revealed comparable results for the social *acceptance* and social *rejection* responsivity analyses, we believe that the rather small voxel size area for social *rejection* responsivity is unlikely to have compromised the statistical power of the analyses.

There are further limitations of our study. First, the CA interview was retrospectively performed with a primary caregiver (Dunn et al., 2011; Goodyer et al., 2010). This might have resulted in underreported CA rates and accordingly in a decreased predictive strength of CA (van Harmelen et al., 2016). However, the time intervals of the CAMEEI (early, middle, and late childhood) enhanced recall and report accuracy of CA, and decreased the impact of recency effects (Dunn et al., 2011). As caregiver reports on CA are found to relate slightly differentially to later mental distress than self-reported CA (Newbury et al., 2018), future studies may want to repeat the analyses either with self-reported CA or ideally with both report forms. Second, friendship and family support were not assessed prior to CA. Therefore, it cannot be determined whether the adolescents with a history of CA already had higher friendship levels prior to the CA experience. Third, the ROOTS sample is wealthier than the average UK population (Goodyer et al., 2010), and in terms of SES, our subsample did not differ from the remaining ROOTS sample, indicating that the generalizability of our results might be restricted to prosperous populations. Fourth, our sample reported mainly mild to moderate CA experiences (Walsh et al., 2014). Future studies are needed to examine the studied relationships in samples that report more severe CA experiences. Similarly, it may also be of interest to investigate the studied relationships in clinical, nonresilient, samples. Fifth, a subset of the CA group had experienced mental health problems in the past, and although at the time of scanning our CA group was characterized by mental health resilience, it is not clear whether these individuals would have similar brain responsivity to social rejection if we had assessed them at a time when they did experience mental health problems. Unfortunately, our sample is not powered to examine whether the effects were similar or distinct in those with versus without previous mental health problems, as this would result in a sample of only 15 adolescents with a history of CA who had no lifetime mental health problems. Therefore, our findings are restricted to current mental health resilience at the time of the social rejection assessment.

To the best of our knowledge, this is the first study to show that adolescents with CA and normative mood levels have more adolescent friendship support and seem to have normal mood and neural responses to social rejection.

## Supplementary material

For supplementary material accompanying this paper visit https://doi.org/10.1017/S0954579419000178. click here to view supplementary material

## Data

The article is based on the first author's embargoed Research Master Thesis (Leiden University Repository). The data that support the findings of this study is derived from the ROOTS study, for details see Goodyer et al. (2010; *International Journal of Epidemiology*: https://doi.org/10.1093/ije/dyp173), as well as from a ROOTS substudy called Cambridge Brain Works Study, for details see Walsh et al. (2012; *NeuroImage*: https://doi.org/10.1016/j.neuroimage.2012.07.067), conducted at the University of Cambridge, Department of Psychiatry, and the Medical Research Council Cognition and Brain Sciences Unit, Cambridge. The data are not publicly available as our participants did not give informed consent for their measures to be made publicly available.
