# Supplementary material for: Mood and neural responses to social rejection do not seem to be altered in resilient adolescents with a history of adversity
Source: Dev Psychopathol. 2020 May;32(2):411–23. doi: 10.1017/S0954579419000178 (PMC7260057; doi:10.1017/S0954579419000178)
Supplement: Supplementary file 1 [file S0954579419000178sup001.pdf]

Appendix A. Childhood Adversity Measures

As the Cambridge Early Experiences Interview (CAMEEI; Dunn et al., 2011; Goodyer, Croudace, Dunn, Herbert, & Jones, 2010) is a semi-structured interview, all CA questions had a "main question" and several "sub-questions". The items of the CAMEEI that were used for the present study were clustered into four categories (a) family discord, (b) sexual abuse, (c) physical abuse, and/or (d) emotional abuse. Family discord was specified as conflict and/or incidental violence within the family, as well as lack of engagement and communication within the family (clustered in mild, moderate and severe). Importantly, only adolescents with a history of family discord that was classified as having a significant impact on daily life (for details see Table 1) were included in the CA group. In the Table below we tabulate the "main questions".

**Topic**	**"Main question(s)" (if applicable followed up by several sub-questions)**
Family discord	Have there been times when family members really haven't got on together?<br>Significant impact on daily life:<br>0. little/no significant impact on family life.<br>1. significant impact on family life. Examples may be: parent/s may have struggled to keep household going or suffered depression or anxiety response to the separation, daily care of children suffered, children may have missed school, been left to own devices or spent some time living elsewhere.
Sexual abuse	As far as you know, did [child's name] suffer sexual maltreatment? (This may have involved sexual touching, exposure, penetration or anything else sexual. It may have involved someone trusted, like a teacher or friend, someone within the family, or a complete stranger.)
Physical abuse	As far as you know, did [child's name] suffer physical maltreatment? (Physical abuse may have involved punching or kicking, scratching, slapping.)
Emotional abuse	As far as you know, did [child's name] suffer emotional maltreatment? (By emotional abuse we mean imposing emotional punishment rather than physical, e.g. withdrawing affection, enforcing silence, isolation, emotional blackmail, humiliation etc.)

Appendix B. Descriptive Measures

B.1. Socio-Economic Status (SES)

The ACORN (A Classification of Residential Neighborhoods (http://www.caci.co.uk); Morgan & Chinn, 1983) is a geodemographic index for the United Kingdom, which classifies SES into five categories using post-codes as indicators. Due to statistical reasons we merged the five clusters into three broader categories: (I) Wealthy achievers and urban prosperity, (II) comfortably off, and (III) moderate means and hard-pressed. SES was assessed at age 14.

B.2. Intelligence

To assess intelligence the vocabulary and block design sub-tests of the Wechsler Abbreviated Scale of Intelligence (WASI; Wechsler, 1999) were utilized and assessed at the scanning occasion (age 18). These two sub-tests of the WASI were found to have the strongest correlation with overall IQ scores, ranging from .8 to .9, and function as adequate IQ proxy (Ryan, 1981).

B.3. Recent Negative Life Events

The Life Events Questionnaire (LEQ; adapted from Goodyer, Herbert, Tamplin, & Altham, 2000) was used to retrospectively assess positive and negative life events for a period of one year, during late adolescence (approximately age 16 to 17). Participants were asked to specify, for all items that have been rated as 'negative', whether they were sad or distressed for more than two weeks. We utilized the self-reported negative events which lasted longer than two weeks as proximal measure for recent negative life events (see Walsh et al., 2012).

B.4. Current and Past Psychiatric Diagnosis

Current and past psychiatric episodes were assessed with the Kiddie Schedule for Affective Disorders and Schizophrenia for School-Age Children – Present and Lifetime Version (K-SADS-PL; Kaufman et al., 1997). Clinical sub-threshold diagnoses and self-harm were also considered as psychiatric history. We included self-harm as indicator for psychiatric history, given that adolescents are found to be at a heightened risk for self-harm behavior (Muehlenkamp, Claes, Havertape, & Plener, 2012; Schmidtke et al., 1996). The K-SADS-PL has been assessed at age 14, age 17, and prior to the fMRI scan at age 18.

B.5. Self-Esteem

The Rosenberg Self-Esteem Scale (RSES; Rosenberg, 1965) was used to assess the adolescents' positive and negative self-image at age 14 and 17. The RSES is a 10 item self-report questionnaire and is reported to have a good internal consistency in a sample of adolescents and young adults (α = .86; Tinakon & Nahathai, 2012).

B.6. Depression Symptoms

The Mood and Feeling Questionnaire (MFQ; Messer, Angold, & Costello, 1995) was used to measure depression symptoms at age 14, age 17, and prior to the fMRI scan at age 18. The MFQ is a 33 item self-report questionnaire and was found to have a good internal consistency for the overall ROOTS sample (α = .93; Messer et al., 1995).

B.7. 5-HTTLPR Genotype

At age 14, we collected saliva samples (Qiagen, Crawley, UK) from which we gathered DNA to eventually classify 'l/l' and 's/s' alleles of the 5-HTTLPR genotype (for details see Walsh et al., 2012, 2014).

B.8. Parental Psychopathology

Parental psychopathology was measured with the MINI Mental State Examination (Sheehan et al., 1998), and was reported by the primary caregiver who also performed the childhood adversity interview. Parental psychopathology was assessed for the time frame from before the participant's birth (i.e. for biological parents) until the date of assessment (for details see Walsh e al., 2014). Parental psychopathology was assessed at age 14.

Appendix C. Figure 1 Modifications

Figure 1 is, with permission, adapted from Dalgleish et al. (2017; Scientific Reports; can be retrieved from https://doi.org/10.1038/srep42010). In the original article, the Figure was published under the Creative Commons Attribution 4.0 International License. Information about this license can be found in the article itself (Dalgleish et al., 2017; https://doi.org/10.1038/srep42010) or at http://creativecommons.org/licenses/by/4.0/. Some modifications were applied to Figure 1, including: (1) minor layout modifications (e.g. background colors, box sizes, component order, letter fonts); (2) we removed one text box containing the text ('self and other ratings on same attributes (based on video feedback)'); (3) minor rephrasing of some text components (e.g. from 'hyperscanning environment' to 'scanning environment' or from 'fMRI task trial x 36' to 'fMRI task 36 trials').

Appendix D. Results Table of the Exploratory Analyses

Single Mediation Models of Current Family and Friendship Support as Mediators for the Relationship between Childhood Adversity and Neural Responses to Social Rejection Feedback

	Indep. Var.	Dep. Var.	est	SE	*z*	*p*(>|*z*|)	CI low	CI up
		Friendship Support and AI Responsivity						
c′ path	CA	AI responsivity	.06	.15	.38	.70	-.24	.35
a path	CA	Friendship	.26	.13	2.06	**.04***	.01	.51
b′ path	Friendship	AI responsivity	-.07	.13	- .55	.58	-.32	.18
a*b			-.02	.04	- .49	.63	-.09	.06
a*b + c′			.04	.13	.29	.77	-.22	.30
		Family Support and AI Responsivity						
c′ path	CA	AI responsivity	.06	.13	.48	.63	-.19	.31
a path	CA	Fam. sup.	-.24	.13	-1.83	.07	-.50	.02
b′ path	Fam. sup.	AI responsivity	.09	.15	.65	.52	-.19	.38
a*b			-.02	.04	- .62	.53	-.09	.05
a*b + c′			.04	.13	.29	.77	-.22	.30
		Friendship Support and dACC Responsivity						
c′ path	CA	dACC responsivity	-.06	.14	- .43	.67	-.33	.21
a path	CA	Friendship	.26	.13	2.06	**.04***	.01	.51
b′ path	Friendship	dACC responsivity	-.07	.11	- .65	.52	-.28	.14
a*b			-.02	.03	- .58	.56	-.08	.04
a*b + c′			-.08	.13	- .59	.56	-.34	.18
		Family Support and dACC Responsivity						
c′ path	CA	dACC responsivity	-.06	.13	- .45	.65	-.32	.20
a path	CA	Fam. sup.	-.24	.13	-1.83	.07	-.50	.02
b′ path	Fam. sup.	dACC responsivity	.08	.15	.52	.61	-.21	.36

a*b	-.02	.04	- .51	.61	-.09	.05
a*b + c’	-.08	.13	- .59	.56	-.34	.18

Note. Indep. Var. = independent variable, Dep. Var. = dependent variable, *SE* = standard error, CA = childhood adversity, Friendship = Friendship support, Fam. sup. = Family support, c’ path = direct effect, a*b = indirect effect, a*b + c’ = total effect.

Appendix E. Variable Correlation Matrices

Whole sample

		Friendship support			Family support			Outcomes		
		Age 14	Age 17	Age 18	Age 14	Age 17	Age 18	AI	ACC	mood
	CA	0.30	0.17	0.26	-0.18	-0.25	-0.24	0.04	-0.08	-0.24
Friend support	Age 14	1.00	0.48	0.29	0.18	-0.14	-0.15	0.04	-0.27	0.03
	Age 17	0.48	1.00	0.67	0.43	0.08	0.02	0.13	0.05	0.03
	Age 18	0.29	0.67	1.00	0.36	0.19	0.20	-0.06	-0.08	0.04
Family support	Age 14	0.18	0.43	0.36	1.00	0.54	0.31	0.04	-0.10	0.00
	Age 17	-0.14	0.08	0.19	0.54	1.00	0.46	-0.02	0.17	0.06
	Age 18	-0.15	0.02	0.20	0.31	0.46	1.00	0.08	0.09	0.26
Outcomes	AI	0.04	0.13	-0.06	0.04	-0.02	0.08	1.00	0.57	0.20
	ACC	-0.27	0.05	-0.08	-0.10	0.17	0.09	0.57	1.00	0.13
	mood	0.03	0.03	0.04	0.00	0.06	0.26	0.20	0.13	1.00

Note. CA = childhood adversity, AI = anterior Insula, ACC = anterior cingulate cortex, mood = negative (vs neutral) mood responsivity during social rejection.

Female sample

		Friendship support			Family support			Outcomes		
		Age 14	Age 17	Age 18	Age 14	Age 17	Age 18	AI	ACC	mood
	CA	0.41	0.31	0.29	-0.34	-0.52	-0.26	-0.16	-0.12	0.23
Friend support	Age 14	1.00	0.61	0.29	0.23	-0.22	-0.12	-0.05	-0.30	0.06
	Age 17	0.61	1.00	0.73	0.42	0.12	0.17	0.25	0.01	0.16
	Age 18	0.29	0.73	1.00	0.38	0.02	0.16	-0.07	-0.12	-0.03
Family sup-	Age 14	0.23	0.42	0.38	1.00	0.61	0.63	0.15	-0.04	-0.07
	Age 17	-0.22	0.12	0.02	0.61	1.00	0.72	0.19	0.13	0.11

port	Age 18	-0.12	0.17	0.16	0.63	0.72	1.00	0.05	0.08	0.11
Out-comes	AI	-0.05	0.25	-0.07	0.15	0.19	0.05	1.00	0.75	0.29
	ACC	-0.30	0.01	-0.12	-0.04	0.13	0.08	0.75	1.00	0.37
	mood	0.06	0.16	-0.03	-0.07	0.11	0.11	0.29	0.37	1.00

Note. CA = childhood adversity, AI = anterior Insula, ACC = anterior cingulate cortex, mood = negative (vs neutral) mood responsivity during social rejection.

Male sample

		Friendship support			Family support			Outcomes		
		Age 14	Age 17	Age 18	Age 14	Age 17	Age 18	AI	ACC	mood
	CA	0.22	0.06	0.21	-0.02	-0.16	-0.31	0.21	-0.04	-0.49
Friend sup-port	Age 14	1.00	0.33	0.36	0.13	-0.01	-0.13	0.12	-0.24	0.00
	Age 17	0.33	1.00	0.71	0.48	0.12	-0.01	0.01	0.07	-0.08
	Age 18	0.36	0.71	1.00	0.36	0.26	0.13	-0.05	-0.05	0.14
Family sup-port	Age 14	0.13	0.48	0.36	1.00	0.50	0.15	-0.06	-0.17	0.04
	Age 17	-0.01	0.12	0.26	0.50	1.00	0.22	-0.18	0.27	0.12
	Age 18	-0.13	-0.01	0.13	0.15	0.22	1.00	0.09	0.11	0.38
Out-comes	AI	0.12	0.01	-0.05	-0.06	-0.18	0.09	1.00	0.41	0.17
	ACC	-0.24	0.07	-0.05	-0.17	0.27	0.11	0.41	1.00	0.01
	mood	0.00	-0.08	0.14	0.04	0.12	0.38	0.17	0.01	1.00

Note. CA = childhood adversity, AI = anterior Insula, ACC = anterior cingulate cortex, mood = negative (vs neutral) mood responsivity during social rejection.

Appendix F. Power Considerations for Testing Moderation Effects

Meta-analytic research suggests that interaction effects in social sciences require strong analytic power. Champoux and Peters (1987), for instance, investigated 23 moderation analyses and revealed that interaction effects account on average for about 3.2 percent of the outcome measure (*M* increase in R^2 = .032). Likewise, Aguinis and colleagues (2005) revealed an average effect size of .01 (f^2) for the 261 investigated interaction effects. We calculated that a moderation analysis with an alpha of .05, a power of .80, a moderately strong main effect of the CA and the support predictor together (f^2 = .15 ≈ R^2 = .13), and a .032 increase in explained variance through the interaction effect (*M* increase in R^2 = .032), would have required 208 participants (f2 = .038; conducted in G*Power; Faul, Erdfelder, Lang, & Buchner, 2007). Even if we would have expected that the main effects of CA and the support predictor together would have a large effect (f^2 = .35 ≈ R^2 = .259) and the interaction effect would again lead to an .032 increase in explained variance (*M* increase in R^2 = .032), we would have required 176 participants (f2 = .045; conducted in G*Power; Faul et al., 2007). Thus, as (1) neither the main effect of CA, nor the main effect of the support variables on brain responses to social rejection revealed significance, and as (2) our power analyses indicated that our sample size would not have been sufficient to detect interaction effects, we considered it for the current study inappropriate to analyze moderation effects.

References

Aguinis, H., Beaty, J. C., Boik, R. J., & Pierce, C. A. (2005). Effect Size and Power in Assessing Moderating Effects of Categorical Variables Using Multiple Regression: A 30-Year Review. *Journal of Applied Psychology*, *90*(1), 94–107. https://doi.org/10.1037/0021-9010.90.1.94

Champoux, J. E., & Peters, W. S. (1987). Form, effect size and power in moderated regression analysis. *Journal of Occupational Psychology*, *60*, 243–255. https://doi.org/10.1111/j.2044-8325.1987.tb00257.x

Dalgleish, T., Walsh, N. D., Mobbs, D., Schweizer, S., Van Harmelen, A. L., Dunn, B., … Stretton, J. (2017). Social pain and social gain in the adolescent brain: A common neural circuitry underlying both positive and negative social evaluation. *Scientific Reports*, *7*, 1–8. https://doi.org/10.1038/srep42010

Dunn, V. J., Abbott, R. A., Croudace, T. J., Wilkinson, P., Jones, P. B., Herbert, J., & Goodyer, I. M. (2011). Profiles of family-focused adverse experiences through childhood and early adolescence: The ROOTS project a community investigation of adolescent mental health. *BMC Psychiatry*, *11*(1), 109. https://doi.org/10.1186/1471-244X-11-109

Faul, F., Erdfelder, E., Lang, A.-G., & Buchner, A. (2007). G*Power 3: A flexible statistical power analysis program for the social, behavioral, and biomedical sciences. *Behavior Research Methods*, *39*(2), 175–191. https://doi.org/10.3758/BF03193146

Goodyer, I. M., Croudace, T., Dunn, V., Herbert, J., & Jones, P. B. (2010). Cohort Profile: Risk patterns and processes for psychopathology emerging during adolescence: the ROOTS project. *International Journal of Epidemiology*, *39*, 361–369. https://doi.org/10.1093/ije/dyp173

Goodyer, I. M., Herbert, J., Tamplin, A., & Altham, P. M. E. (2000). Recent life events, cortisol, dehydroepiandrosterone and the onset of major depression in high-risk adolescents. *British Journal of Psychiatry*, *177*, 499–504. https://doi.org/10.1192/bjp.177.6.499

Kaufman, J., Birmaher, B., Brent, D., Rao, U., Flynn, C., Moreci, P., … Ryan, N. (1997). Schedule for Affective Disorders and Schizophrenia for School-Age Children-Present and Lifetime Version (K-SADS-PL): Initial reliability and validity data. *Journal of the American Academy of Child and Adolescent Psychiatry*, *36*(7), 980–988. https://doi.org/10.1097/00004583-199707000-00021

Messer, S. C., Angold, A., & Costello, E. J. (1995). Development of a Short Questionnaire for Use in Epidemiological Studies of Depression in Children and Adolescents: Factor Composition and Structure across Development. *International Journal of Methods in Psychiatric Research*, *5*, 251–262.

Morgan, M., & Chinn, S. (1983). ACORN group, social class, and child health. *Journal of Epidemiology and Community Health*, *37*, 196–203.

Muehlenkamp, J. J., Claes, L., Havertape, L., & Plener, P. L. (2012). International prevalence of adolescent non-suicidal self-injury and deliberate self-harm. *Child and Adolescent Psychiatry and Mental Health*, *6*, 10. https://doi.org/10.1186/1753-2000-6-10

Rosenberg, M. (1965). *Society and the Adolescent Self-Image*. Princeton, NJ: Princeton University Press.

Ryan, J. J. (1981). Clinical Utility of a WISC-R Short Form. *Journal of Clinical Psychology*, *37*(2), 389–391.

Schmidtke, A., Bille-Brahe, U., DeLeo, D., Kerkhof, A., Bjerke, T., Crepet, P., … Sampaio-Faria, J. G. (1996). Attempted suicide in Europe: rates, trends and sociodemographic

characteristics of suicide attempters during the period 1989-1992. Results of the WHO/EURO Multicentre Study on Parasuicide. *Acta Psychiatrica Scandinavica*, *93*, 327–338.

Sheehan, D. V., Lecrubier, Y., Harnett Sheehan, K., Amorim, P., Janavs, J., Weiller, E., … Dunbar, G. C. (1998). The Mini-International Neuropsychiatric Interview (M.I.N.I.): The development and validation of a structured diagnostic psychiatric interview for DSM-IV and ICD-10. *Journal of Clinical Psychiatry*, *59*, 22–33. https://doi.org/10.1016/S0924-9338(99)80239-9

Tinakon, W., & Nahathai, W. (2012). A comparison of reliability and construct validity between the original and revised versions of the Rosenberg Self-Esteem Scale. *Psychiatry Investigation*, *9*, 54–58. https://doi.org/10.4306/pi.2012.9.1.54

Walsh, N. D., Dalgleish, T., Dunn, V. J., Abbott, R., St Clair, M. C., Owens, M., … Goodyer, I. M. (2012). 5-HTTLPR-environment interplay and its effects on neural reactivity in adolescents. *NeuroImage*, *63*, 1670–1680. https://doi.org/10.1016/j.neuroimage.2012.07.067

Walsh, N. D., Dalgleish, T., Lombardo, M. V., Dunn, V. J., van Harmelen, A.-L., Ban, M., & Goodyer, I. M. (2014). General and specific effects of early-life psychosocial adversities on adolescent grey matter volume. *NeuroImage: Clinical*, *4*, 308–318. https://doi.org/10.1016/j.nicl.2014.01.001

Wechsler, D. (1999). *Wechsler Abbreviated Scale of Intelligence*. Harcourt Brace & Company, NY: The Psychological Corporation.
